# Spatially resolving how cMyBP-C phosphorylation and haploinsufficiency in porcine and human myofibrils affect β-cardiac myosin activity

**DOI:** 10.1085/jgp.202413628

**Published:** 2025-07-07

**Authors:** Matvey Pilagov, Sonette Steczina, Ateeqa Naim, Michael Regnier, Michael A. Geeves, Neil M. Kad

**Affiliations:** 1 School of Natural Sciences, University of Kent, Canterbury, UK; 2 Department of Bioengineering, University of Washington, Seattle, WA, USA

## Abstract

β-cardiac myosin mediates cardiac muscle contraction within the sarcomere by binding to the thin filament in an ATP-powered reaction. This process is highly regulated on a beat-to-beat basis by calcium interactions with the thin filament, but also contractile force is highly regulated by controlling the number of myosins available, resulting in a dynamic reserve. Our goal was to examine the size of this reserve and how it is modulated by cardiac myosin binding protein-C (cMyBP-C). We used single-molecule imaging to determine myosin activity with high spatial resolution by measuring fluorescently tagged ATP molecules binding to and releasing from myosins within the cardiac sarcomere. Three myosin ATPase states were detected: the fastest species was consistent with nonspecific ATP binding to myosin’s surface, and the slower two species were consistent with the previously identified DRX and SRX states. The former represents myosins in a state ready to interact with the thin filament, and the latter in a cardiac reserve state with slowed ATPase. We found the cardiac reserve was 46% across the whole sarcomere in porcine myofibrils. Subdividing into the P-, C-, and D-zones revealed the D-zone has the smallest population of reserve heads (44%). Treatment with PKA that phosphorylates cMyBP-C led to a 16% reduction of reserve in the C-zone (where cMyBP-C is found) and a 10% reduction in the P-zone, with an unexpected 15% increase in the D-zone. Interestingly, the changes in SRX myosin head distribution by PKA phosphorylation of cMyBP-C across each subsarcomeric zone mirror the changes we identified in human cardiac myofibrils isolated from a hypertrophic cardiomyopathy patient mutation (*MYBPC3*-c.772G>A) that exhibits cMyBP-C haploinsufficiency. These results provide novel insights into how the C-zone functions in both porcine and human β-cardiac myosin–containing thick filaments, revealing a possible compensatory change in the D-zone upon altered cMyBP-C phosphorylation and/or haploinsufficiency.

## Teaser

Single-molecule imaging provides a high spatial resolution of fluorescent ATP binding to myosin within cardiac myofibrils. This is used to determine how cMyBP-C affects the reserve of cardiac myosins used to modulate force. Here, we study PKA phosphorylation and a haploinsufficiency-causing mutation from human tissue.

## Introduction

Cardiac muscle contraction is dynamically regulated to ensure rapid and constant adaptation to the changing physiological demands placed on the heart. The fundamental contractile organelle in cardiac muscle is the myofibril, which is comprised of a tandem array of sarcomeres. Sarcomeres consist of myosin-containing thick filaments and actin-containing thin filaments, both of which are regulated to control the timing and force of contraction. Thin filament regulation is mediated by calcium binding to thin filament–associated regulatory proteins and has been well characterized (Gordon et al., 2000), although some questions remain. However, our understanding of thick filament regulation is still being developed. The current view of thick filament regulation suggests that myosins adopt two biochemical states under relaxed conditions, one poised to interact with actin termed the disordered-relaxed (DRX) state, and a second super-relaxed (SRX) state that constitutes a reserve population of myosins with significantly reduced ATPase activity (Stewart et al., 2010; Hooijman et al., 2011; Nogara et al., 2016). The SRX state is thought to act as a reserve population, recruited when the heart needs stronger contraction but to save energy when they are not immediately required.

Recent high-resolution structural studies have provided valuable insights into the complex environment of the sarcomere (Tamborrini et al., 2023; Dutta et al., 2024). These complement classical studies (Craig and Offer, 1976; Tonino et al., 2019; Bennett and Gautel, 1996) that have used counterstaining to define regions of the thick filament based on the presence of cMyBP-C and titin. The P-zone (0–159 nm) is closest to the M-line (0 nm), the C-zone (160–500 nm) is marked by the clear presence of nine bands of myosin binding protein-C (MyBP-C), and the D-zone (501–800 nm) accounts for the remainder—occupying approximately the last third of the thick filament (Tonino et al., 2019). Interestingly, despite the use of compounds thought to promote myosin into a stabilized, folded-back (interacting-heads motif) state, the D-zone was not resolved in these structural studies. This suggests that the ancillary myosin binding proteins could assist in generating order on the thick filament backbone. Myosin’s biochemical activity is mediated by load (Linari et al., 2015; Fusi et al., 2016), by direct calcium binding to both thin and thick filaments (Ma et al., 2022; Lehman et al., 1973; Bremel and Weber, 1975; Podlubnaya et al., 1999; Podlubnaya et al., 2000a; Podlubnaya et al. 2000b), and by phosphorylation (Heling et al., 2020; Colson et al., 2010; Levine et al., 1996; Ponnam et al., 2019; McNamara et al., 2019). Phosphorylation affects several proteins in the sarcomere; in particular, rapid phosphorylation of MyBP-C by protein kinase-A (PKA) (activated by the β-adrenergic response pathway) has been shown to result in the derepression of myosin activity (Colson et al., 2010; Ponnam et al., 2019; Tong et al., 2008; Previs et al., 2016). Through such a mechanism, the ratio of SRX to DRX myosins in the myofibril can be altered, which is crucial to changing the force that can be generated. Both the dynamics of the SRX/DRX equilibrium (Mohran et al., 2024) and the distribution of the SRX/DRX states along the thick filament remain unclear. Consistent with MyBP-C affecting this ratio (McNamara et al., 2019; McNamara et al., 2016), recent attempts to localize the distribution of SRX and DRX myosins within the sarcomere have shown that the SRX state predominates in the C-zone of skeletal muscle (Nelson et al., 2020; Pilagov et al., 2023) and in mouse α-cardiac myosin (Nelson et al., 2023). However, to date, there has been no investigation of the β-cardiac isoform that predominates in the ventricles of large mammals including humans and pigs. The pathophysiological importance of understanding how phosphorylation affects activity is highlighted by the observation that cardiac myosin binding protein-C (cMyBP-C) is one of the two most prevalent locations for mutations associated with hypertrophic cardiomyopathy (HCM) (Konno et al., 2010). Disease-specific changes associated with HCM *MYBPC3* mutations have led to reduced cMyBP-C production from one allele and haploinsufficiency within the thick filament (Carrier et al., 2015; Barefield et al., 2015; Suay-Corredera and Alegre-Cebollada, 2022; Pioner et al., 2023; Steczina et al., 2024; Glazier et al., 2019). The impact of such haploinsufficiency on thick filament regulation has so far only been studied without spatial resolution, revealing a shift toward more DRX myosins (Toepfer et al., 2019). Consistent with this, complete ablation of cMyBP-C in a transgenic rodent model has been linked to a structural release of myosin heads from the thick filament backbone (Colson et al., 2007), as well as an enhanced biochemical phenotype characterized by a global shift away from the SRX state (McNamara et al., 2016; Toepfer et al., 2019).

In this study, we sought to establish the zonal distribution of DRX and SRX myosins for both porcine- and human heart–derived myofibrils, with an aim to understand how cMyBP-C alters the cardiac reserve. To achieve this, we employed a single-molecule approach to image the ATPase activity of individual myosins within the myofibrillar lattice under relaxed conditions. For porcine left ventricular (LV) cardiac myofibrils, we studied PKA phosphorylation of cMyBP-C, and for human septal myectomy myofibrils, we investigated the HCM mutation (*MYBPC3*-c.772G>A) from human patient–derived tissue that results in cMyBP-C haploinsufficiency. A recent investigation of this HCM mutation revealed ∼30% cMyBP-C haploinsufficiency across three patients with this mutation (Pioner et al., 2023). Both elevated cMyBP-C phosphorylation and HCM-linked cMyBP-C haploinsufficiency resulted in prominent zonal effects. Our results suggest that these alterations to cMyBP-C lead to elevated ATPase activity in the C-zone and a partially compensatory reduction in ATPase activity in the D-zone. These findings suggest the possibility of a negative cooperativity effect along the thick filament, a phenomenon not observed in skeletal muscle (Nelson et al., 2020; Pilagov et al., 2023), and underscores the value of studying isoform-specific thick filament regulation with subsarcomeric resolution.

## Materials and methods

No live animals were used in these studies. Muscle tissue was collected in accordance with the U.K. Animals (Scientific Procedures) Act 1986 and associated guidelines.

### Dissection and storage of porcine heart samples

Immediately following excision, the heart of a freshly euthanized adult farm pig was submerged in ice-cold cardioplegic solution (5.5 mM glucose, 0.5 mM MgSO_4_, 24 mM KCl, 20 mM NaHCO_3_, 109 mM NaCl, 0.9 mM H_2_NaO_4_P, 1.8 mM CaCl_2_, 0.01% NaN_3_, pH 7.4). While remaining in solution, LV trabecula samples were extracted from the heart and cut into ∼5-mm-thick pieces, immediately flash-frozen, and kept at −80°C for long-term storage.

### Dissection and storage of human myectomy samples

Myectomy samples used for this study included HCM sarcomere mutation-negative cardiac tissue used as control samples (*N* = 2, 37 and 45 years at time of myectomy, males) and HCM sarcomere mutation-positive cardiac tissue confirmed via sequencing to harbor the *MYBPC3*-c.772G>A mutation (*N* = 1, 33 years at time of myectomy, male). Human cardiac tissue samples were isolated during surgical myectomy to relieve obstruction of the LV outflow tract. Tissue was prepared as previously described (Pioner et al., 2023). Briefly, surgical samples were washed in ice-cold cardioplegic solution (140 mM glucose, 100 mM mannitol, 10 mM taurine, 50 mM KH_2_PO_4_, 8 mM MgSO_4_, 10 mM HEPES, 5 mM adenosine, pH 7.4, with KOH) and then cut into smaller sections to be flash-frozen and stored long term at −80°C.

### Myofibril isolation and fluorescent staining

Myofibril isolations were carried out as previously described in Pilagov et al. (2023) with adaptations for cardiac tissue. Briefly, a single porcine LV trabecula sample or human cardiac tissue sample was rapidly thawed in chilled Prep buffer (20 mM MOPS, 132 mM NaCl, 5 mM KCl, 4 mM MgCl_2_, 5 mM EGTA, 10 mM NaN_3_, 5 mM DTT, 20 mM 2,3-butanedione monoxime [BDM], protease inhibitor cocktail [A32965; Thermo Fisher Scientific], pH 7.1) (Vikhorev et al., 2016) and cut into 2- to 3-mm-thick strips. Samples were secured to a Sylgard PDMS-based petri dish with tungsten rods at both ends of the tissue, and Prep buffer was replaced with Permeabilization buffer (Prep buffer + 0.5% Triton X-100). Samples were permeabilized overnight with gentle agitation (4°C) and then rinsed three times with Prep buffer to remove Triton X-100. Permeabilized samples were cut down to ∼1-mm-thick strips, then homogenized in Prep buffer using a TissueRuptor II at the slowest speed for 10 s twice with a 1-min rest on ice in between. The concentration of myofibril preparations was kept constant by targeting an optical density of ∼0.4 at 600 nm at the end of the preparation.

For fluorescent tagging of Z-disks, isolated myofibrils were incubated for 1.5 h at 4°C on a rotator with 11 nM anti-α-actinin mouse antibody (A7811; Sigma-Aldrich), 5.5 nM Alexa 488 goat anti-mouse IgG (A11001; Thermo Fisher Scientific), and 1 mg/ml bovine serum albumin, to avoid nonspecific antibody binding.

### Imaging conditions

Microfluidic imaging chambers were constructed and coated with 15 µg/ml >300 KDa poly-L-lysine (PLL, Sigma-Aldrich) on the day of imaging as described previously (Pilagov et al., 2023) and rinsed with 100 μl Prep buffer. Fluorescently tagged myofibrils were pipetted into the imaging chamber and incubated for 30 min at 4°C to allow adhesion of myofibrils to the PLL-coated surface. Excess nonadherent myofibrils were flushed out with two washes of 100 μl No BDM Buffer (Prep buffer without BDM) with a 1-min rest at 4°C in between. Finally, 100 μl Imaging buffer (No BDM Buffer plus 1–15 nM Cy3-ATP, 3.27–5 mM ATP, and 2 mM Trolox) was added to the flow chamber and allowed to equilibrate for 10 min prior to imaging. Cy3-ATP was synthesized and kindly provided by Dr. C.P. Toseland (University of Sheffield, Sheffield, UK) (Toseland and Webb, 2011). To ensure no Cy3-ADP contamination, the nucleotide was enzymatically regenerated into Cy3-ATP prior to the experiments, as described previously (Pilagov et al., 2023). All solutions were gently flowed into the imaging chamber at ∼100 μl/min to prevent dislodging of myofibrils from the surface.

### PKA treatment

A subset of porcine cardiac myofibrils were treated for 1 h at 4°C on a rotator with 62.5 U/μl of cAMP-dependent protein kinase, catalytic subunit (P600S; New England Biolabs), NEBuffer for protein kinases (B6022S; New England Biolabs), and 200 µM ATP. PKA treatment was carried out at a lower temperature and for longer than recommended to prevent degradation of myofibrils.

### Image acquisition

Images were acquired at 21°C on a custom-built oblique angle fluorescent microscope as described previously (Desai et al., 2015). For excitation of the Cy3-ATP, a 561-nm diode OBIS LS laser (Coherent) was used at 20 mW and the Alexa 488 anti-α-actinin was excited with an Oxxius 488-nm laser at 18 mW. Images were captured using a Hamamatsu Orca-Flash V.20 sCMOS camera. To prevent fluorescence photobleaching, myofibrils were located using bright-field microscopy. Photobleaching was also ameliorated during image acquisition via the use of stroboscopic illumination as previously described (Pilagov et al., 2023). Stroboscopic illumination resulted in a six-frame repeat pattern consisting of five sequential, 200-ms 561-nm laser Cy3-ATP frames with a dark period of 1,800 ms. The sixth frame was a 200-ms 488-nm illumination during the 1,800-ms dark period of the fifth 561-nm laser cycle to capture an image of the Z-disks. Each sample was imaged for 30 min producing 1,000-frame videos comprised of the Z-disk and Cy3-ATP frames. To prevent ADP and Cy3-ADP accumulation, chambers were used for no longer than 1 h.

### Data analysis

To extract the spatiotemporal data of Cy3-ATP binding events, analysis was carried out using a series of custom-written scripts. Firstly, a Python script divided the 1,000-frame video into two videos, one containing all the ATP frames and the other containing all the Z-disk frames. TrackMate, an ImageJ plugin (Tinevez et al., 2017; Ershov et al., 2022), was utilized for automated tracking and super-localization of fluorescent Cy3-ATP and Z-disks. The following parameters were used to run TrackMate: estimated object diameter for the Laplacian-of-Gaussian detector: 390 nm; linking and gap-closing distances: 100 nm; and gap-closing max frame: 5 frames. All videos were manually checked against the TrackMate output to ensure correct track assignment. If myofibril drift was identified across the duration of a video, this was corrected using a star-map approach (Descriptor_based_registration, 2015, https://github.com/fiji/Descriptor_based_registration) prior to event tracking. Annotated Scripts are available (see Data availability).

**Figure S1.**
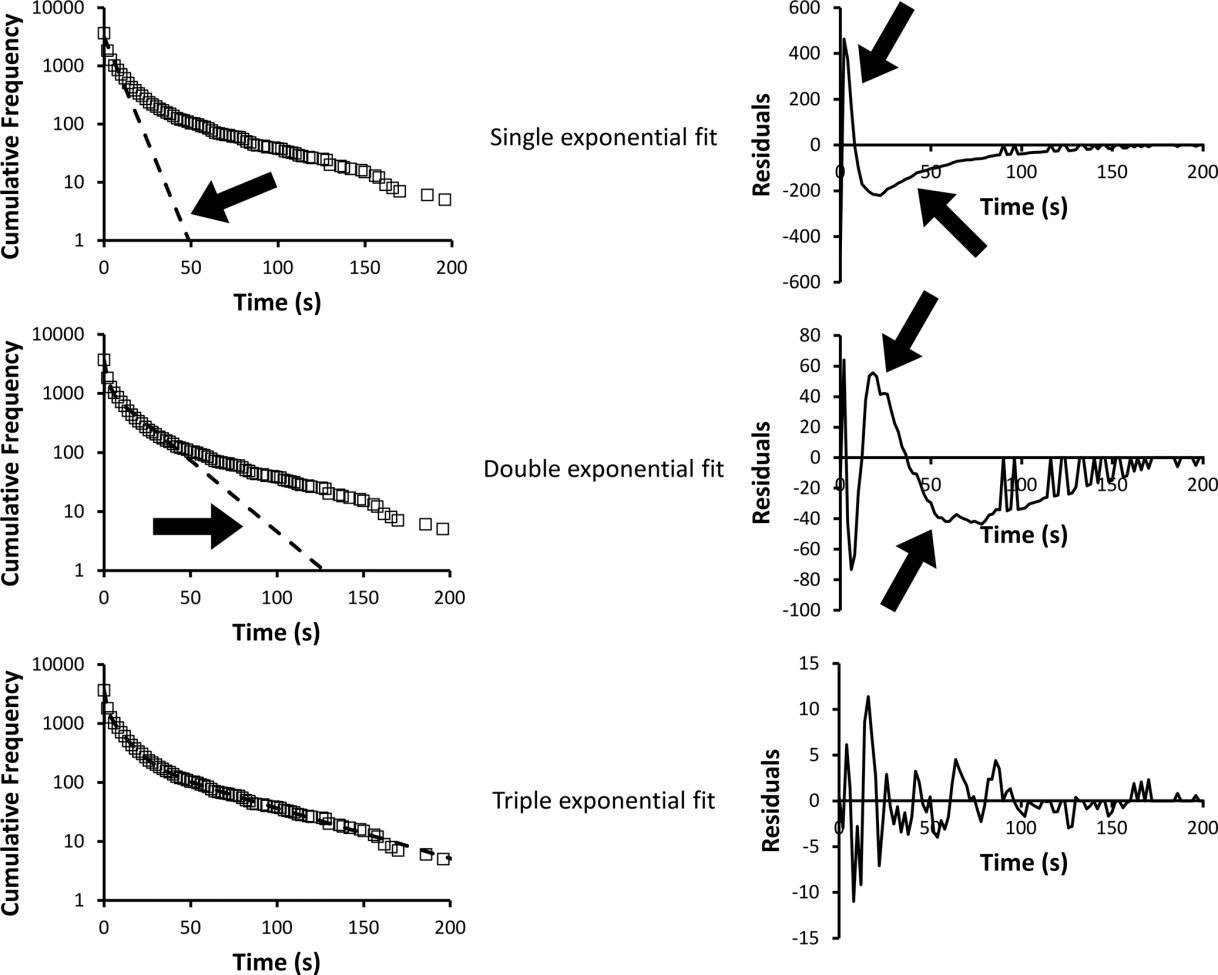
**Residual and cumulative frequency graphs for determining the optimal number of fitted exponentials.** (Left) In this example, untreated all zones data are shown (squares) fitted to an increasing number of exponentials (dashed lines). (Right) For the corresponding fit, we show the residuals (fitted values minus data values). A larger amplitude on the y axis means a poorer fit, and it can be seen that the amplitudes decrease as the number of fitted exponentials increases. However, a clear deviation from the data is seen (arrows on left graphs) and the systematic noise (arrows on right graphs) remains until three exponentials are used. In the latter case, the maximum noise after fitting is <0.5%.

TrackMate provided super-localized X- and Y-positions of Cy3-ATP events and their durations, which were then extracted to Microsoft Excel for further analysis. Based on our previously described method (Pilagov et al., 2023), Z-disk images were used to correct any tilt and as fiducial markers to locate Cy3-ATP positions to zones of the A-band. Spatial precision for both Z-disk and Cy3-ATP events was as follows: X—68.2 nm, Y—135.4 nm and X—28.1 nm, Y—38 nm, respectively (Gelles et al., 1988). Cy3-ATP events were binned into three zones of the A-band: P-zone (0–159 nm), C-zone (160–500 nm), and D-zone (501–800 nm) (Tonino et al., 2019). All distances are given from the M-line, which was estimated as the midpoint between adjacent Z-disks and assumes the A-band is centrally and symmetrically located in the sarcomere. Events outside indicated areas of the A-band were excluded when plotting and fitting cumulative frequency graphs of Cy3-ATP events as performed previously (Pilagov et al., 2023). Cy3-ATP events were plotted as cumulative residence time histograms for both zonally separated events (P-, C-, and D-zones) and events across the whole thick filament (denoted as “all zones” data). Cumulative residence time histograms were fitted using least squares regression (Solver, Microsoft Excel) to three exponentials $\left({A_{t}=A_{1}e}^{-k_{1}t}+{A_{2}e}^{-k_{2}t}+{A_{3}e}^{-k_{3}t}\right)$. Where *A* is the amplitude of the numbered phase, *k* is the rate constant of the numbered phase, and *t* is time. To determine the optimal number of exponentials required to fit these data, an error analysis on the residuals was performed, looking for residuals that showed no systematic differences and possessed a small amplitude (see Fig. S1). For accurate fitting within the subsarcomeric zones, we used two constraints based on the fitting of the all zones data: the sum of the zonal events was constrained to within 20% of that determined for the all zones data, and the rate constants per zone were permitted to vary within 20% of the all zones rate constants. Based on our previous study (Pilagov et al., 2023), the three fitted exponentials corresponded to: (1) nonspecific association of Cy3-ATP with myosin (Ušaj et al., 2021; Amrute-Nayak et al., 2014), (2) Cy3-ATP turnover association with myosin in the DRX state, and (3) Cy3-ATP turnover association with myosin in the SRX biochemical state. Errors for cumulative residence time histogram fits were determined by performing a 1,000-sample bootstrapping with replacement routine using Microsoft Excel. The same constraints on the rate constants as described above were imposed, including for the all zones data. From this distribution of fitted parameters, the 95% confidence intervals (CIs) were calculated. All fitted rate constants are shown in Table S1.

### Phosphoprotein gels

Precast 4–20% gradient Tris-glycine gels (XP04205BOX; Invitrogen) were used to separate cardiac sarcomere proteins of interest. The ladder used was PageRuler Plus Prestained Protein Ladder (26619; Thermo Fisher Scientific). Samples were boiled for 5 min with standard SDS sample loading buffer prior to loading. Gels were stained with Pro-Q Diamond Phosphoprotein Gel Stain (P33300; Thermo Fisher Scientific) to determine the phosphorylation level of cMyBP-C. Gels were subsequently stained with Coomassie gel dye to quantify the total protein per sample. Phosphorylation of cMyBP-C was calculated as a relative change in phosphorylation normalized to loading using the cMyBP-C band from the Coomassie gel. Pro-Q gels were imaged using Syngene G:BOX Chemi XX6 set to Pro-Q imaging setting (green lamp, 605-nm filter), and Coomassie was imaged using a white light setting.

### Statistics

Accuracy for TrackMate super-resolved events for both Cy3-ATP events and Z-disks was established using variance from fixed points in the image as described previously (Gelles et al., 1988; Pilagov et al., 2023). For rate constant amplitudes derived from the cumulative residence time histogram fits, significance was determined as nonoverlap of 95% CI and indicated by asterisks (*). n is the number of Cy3-ATP attachments for the respective zone. N equals the number of biological replicates.

### Online supplemental material

**Figure S2.**
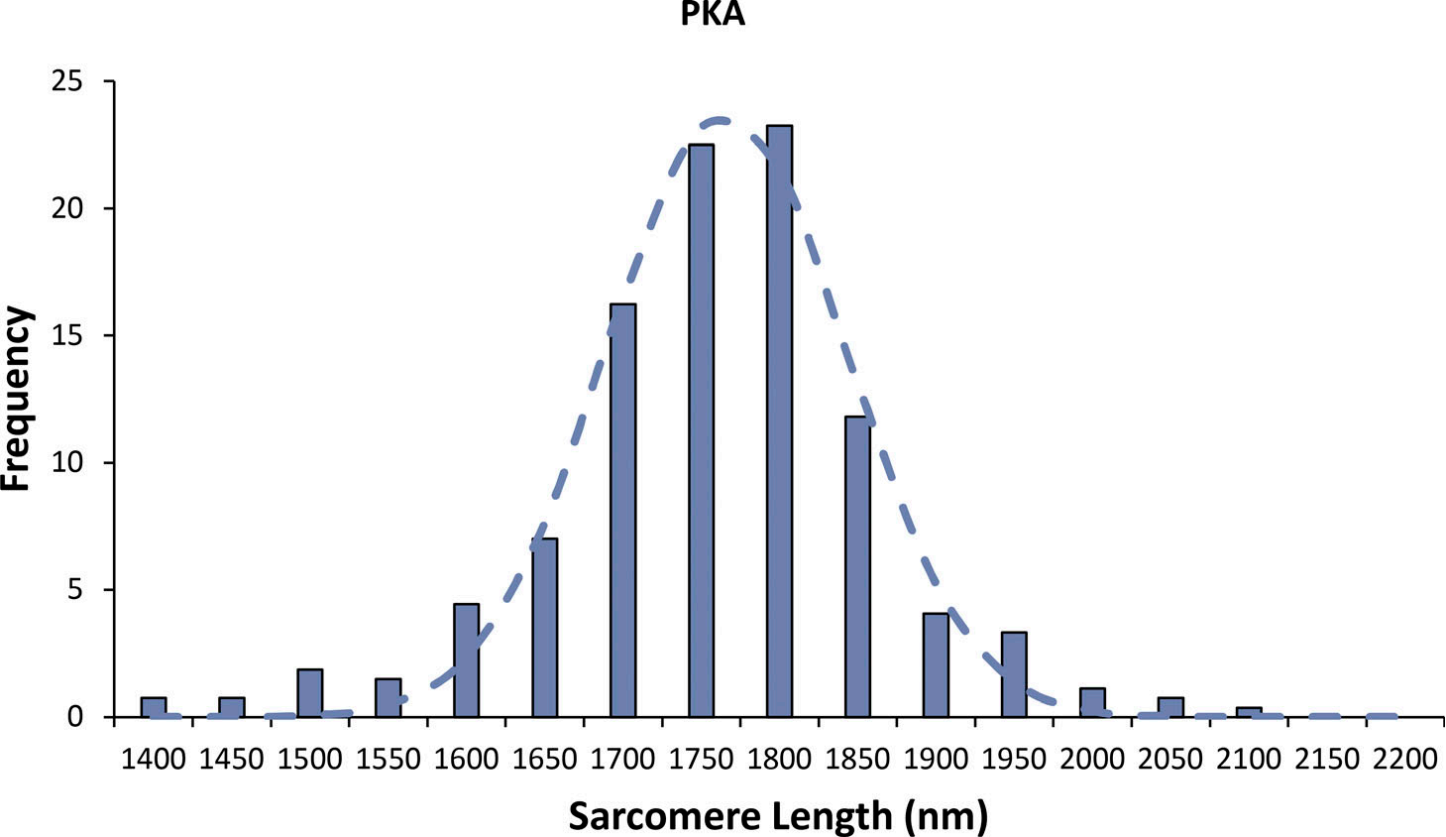
**Distribution of sarcomere lengths for PKA-treated porcine cardiac myofibrils.** Histogram of the distribution of sarcomere lengths for PKA-treated myofibrils fitted to a Gaussian distribution (dotted line) provides a mean and SD 1,766 ± 38.5 nm.

Fig. S1 shows residual and cumulative frequency graphs for determining the optimal number of fitted exponentials. Fig. S2 shows distribution of sarcomere lengths for PKA-treated porcine cardiac myofibrils. Table S1 provides the fitted parameters and errors.

## Results

### Imaging fluorescent ATP lifetimes in relaxed myofibrils

**Figure 1.**
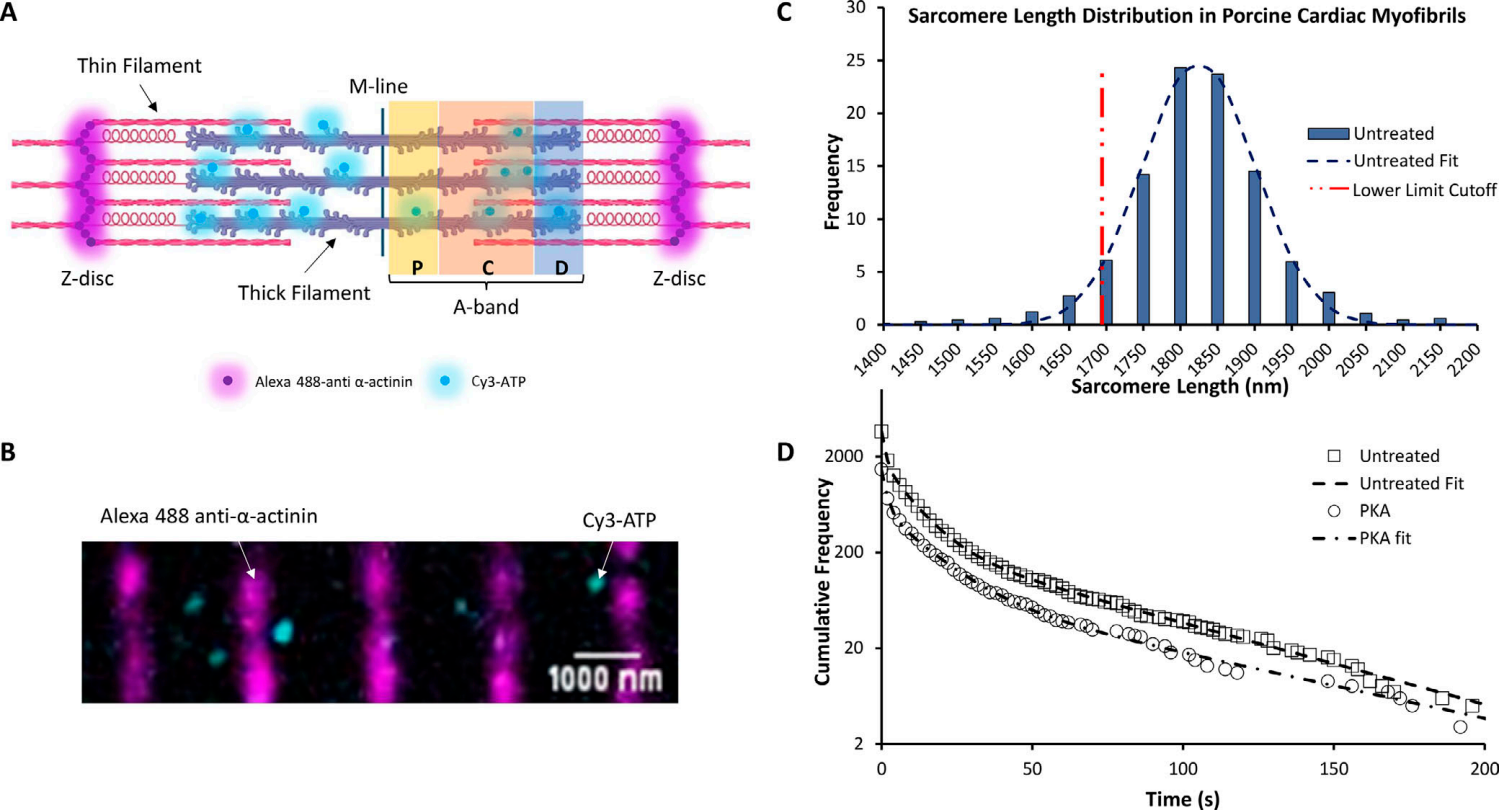
**Single-molecule tracking of fluorescently labeled ATP turnover in porcine cardiac myofibrils. (A)** Schematic representation of a single sarcomere. P-, C-, and D-zones of the thick filament are labeled accordingly. Alexa 488–labeled α-actinin is denoted in magenta, while Cy3-ATP binding to the thick filament is represented as cyan circles. **(B)** Enlarged section of a representative porcine cardiac LV myofibril as observed following the overlay of 561-nm illuminated Cy3-ATP (cyan) and 488-nm illuminated α-actinin–containing Z-disks (magenta). **(C)** Distribution of sarcomere length for untreated porcine myofibrils with a mean of 1.82 µm ± 0.04 SD (*N* = 3 pigs, 647 sarcomeres) at 21°C. Distribution of sarcomere lengths for PKA-treated samples can be found in Fig. S2. The red dotted line indicates the threshold for the smallest sarcomere that was analyzed for all datasets. **(D)** Cumulative residence time histograms showing the distribution of ATP attachment event durations in untreated porcine cardiac myofibrils (circles; *N* = 3 pigs, *n* = 3,638 events) and PKA-treated porcine cardiac myofibrils (squares; *N* = 3 pigs, *n* = 1,463 events). Dashed lines show triple-exponential fits. Note the frequency axis is plotted on a logarithmic scale.

The sarcomere consists of interdigitated thick and thin filaments flanked by α-actinin–containing Z-disks (Fig. 1 A). To determine where in the relaxed sarcomere myosin is actively turning over ATP, we first imaged the positions of the Z-disks as fiducial markers (Fig. 1, A and B). This was achieved using a fluorescently tagged anti-α-actinin–containing antibody (Fig. 1 B, magenta). From such images, we measured sarcomere length by using super-localization analysis (see below); this information was used to select myofibrils with a sarcomere length above 1.7 µm (Fig. 1 C). This ensured that only myofibrils in a clearly relaxed state were used for further analysis.

To determine the activity of individual myosins, attachment durations of Cy3 fluorescently labeled ATP (Cy3-ATP) were measured, previously shown to have no impact on myosin’s activity (Pilagov et al., 2023; Toseland and Webb, 2011; Ušaj et al., 2021). A ∼10^6^-fold excess of nonfluorescent ATP (3.27–5 mM) was used to ensure that the myofibril remained relaxed throughout the experimental duration. However, given that observation times were ∼30 min, it was necessary to include ongoing corrections to the positions of the fiducial markers. Therefore, for every five Cy3-ATP imaging frames, an image of the Z-disks was taken to realign the video. During each Cy3-ATP frame (Fig. 1 B), the position of every fluorescent ATP was tracked and super-localized using the ImageJ plugin, TrackMate. The attachment duration for each bound Cy3-ATP was derived from the video analysis to enable the assignment of Cy3-ATP’s bound lifetime to its location within the sarcomeric A-band. Using the whole thick filament as a starting point, all the lifetimes were combined together as a cumulative residence time histogram (Fig. 1 D, “untreated” data). As with our previous observations in skeletal muscle (Pilagov et al., 2023), three populations best described the observed decay. These three populations fitted to lifetimes consistent with nonspecific ATP binding (1.4 s [95% CI (1.1, 1.5)]; described in Ušaj et al. [2021], Amrute-Nayak et al. [2014]), the DRX state (11.9 s [95% CI (9.8, 12.6)]), and the SRX state (284.3 s [95% CI (167.5, 317.5)]); these lifetimes were photobleach-corrected as described previously (Pilagov et al., 2023). The same imaging and analysis pipeline was used for both porcine cardiac myofibrils and human cardiac myofibrils.

**Figure 2.**
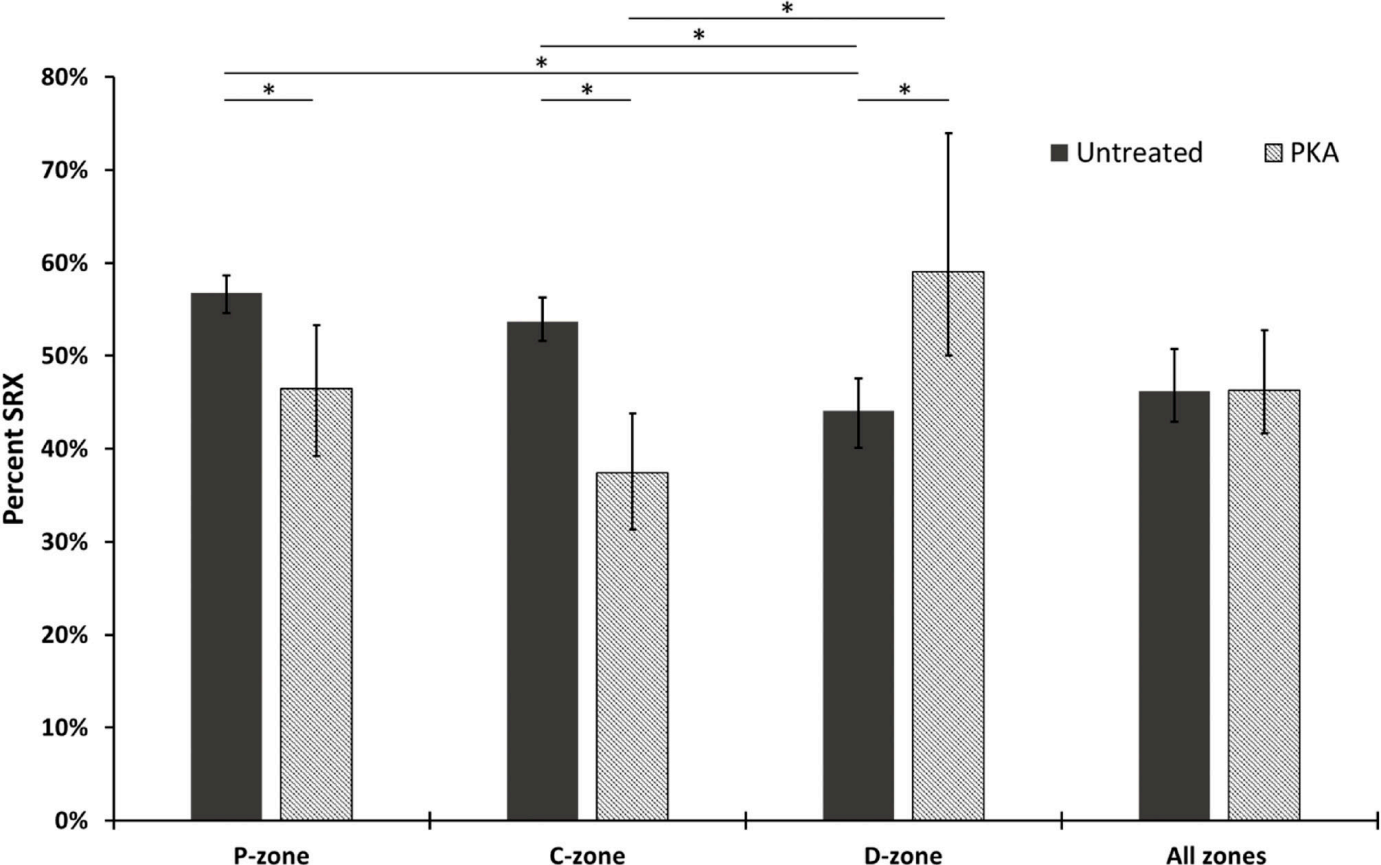
**Subsarcomeric resolution of myosin activity reveals cMyBP-C phosphorylation reduces SRX in the P- & C-zones, compensated for by an increase in the D-zone.** Cy3-ATP turnover rates were calculated as an average across all zones of the sarcomere, as well as individually for the P-, C-, and D-zones. The Cy3-ATP turnover rates per zone were used to calculate the relative population amplitude of SRX myosin heads based on triple-exponential fits. Data were collected from untreated porcine myofibrils (*N* = 3 pigs, *n* = 3,638 events) or myofibrils treated with 62.5 U/μl of PKA (*N* = 3 pigs, *n* = 1,463 events). Data are shown as the mean and 95% CI errors. Significance was calculated using bootstrap analysis and indicated by *. Table 1 provides the values used in the graph. PKA phosphorylation affects the distribution of SRX and DRX in porcine myofibrils.

Using the lifetimes to identify the populations of myosin activity, we were able to calculate the percent population of SRX myosins in the sarcomere. The amplitudes of each phase provided the starting point for estimating the populations. During single-molecule imaging, faster ATP cycling events will be observed more frequently than slower turnover events; therefore, amplitudes were scaled against the observed rate constant to correct for this rate-induced artifact. To achieve this, the ratio of the rate constants (DRX/SRX) was multiplied by the SRX amplitude to calculate a corrected SRX population as previously described (Pilagov et al., 2023). Once corrected, the percent population of SRX for untreated porcine myofibrils was 46.2% (95% CI [50.8, 42.9]) across the thick filament (the nonspecific population of ATP events was not included in these calculations). To identify whether there were differences in the distribution of DRX and SRX myosins along the thick filament, we binned the super-resolution information provided by TrackMate into the P-, C-, and D-zones (Fig. 2 and Table 1). We observed similar percent populations of SRX in the P- and C-zones (56.7% [95% CI (58.7, 54.6)] and 53.7% [95% CI (56.3, 51.6)], respectively); however, the D-zone had a significantly lower percentage of myosins in the SRX state (44.1% [95% CI (47.6, 40.1)]). These results highlight that a more nuanced view of myosin activity within the sarcomere is critical for studies of the thick filament.

**Table 1.** Values for the percentage change in the SRX population across the thick filament for untreated and PKA-treated porcine cardiac myofibrils, as plotted in Fig. 2

	% SRX			
	P	C	D	All zones
Untreated [95% CI]	**56.7** [58.7, 54.6]	**53.7** [56.3, 51.6]	**44.1** [47.6, 40.1]	**46.2** [50.8, 42.9]
*n*	*714*	*1,183*	*1,741*	*3,638*
PKA [95% CI]	**46.5** [53.3, 39.3]	**37.4** [43.8, 31.3]	**59.0** [73.9, 50.1]	**46.3** [52.7, 41.7]
*n*	*224*	*528*	*711*	*1,463*
Absolute Δ SRX (%) [95% CI]	**−10.3** [-3.4, −17.4]	**−16.3** [-9.5, −22.8]	**14.9** [30.5, 5.3]	**0.1** [7.4, −6.1]

Measured values for the percentage SRX are given in bold and 95% CI in square brackets. The absolute difference in SRX as a result of PKA treatment relative to untreated myofibrils is given in the bottom row. The number of events (n) are shown in italics for each area of the thick filament and include nonspecific ATP binding events.

**Figure 3.**
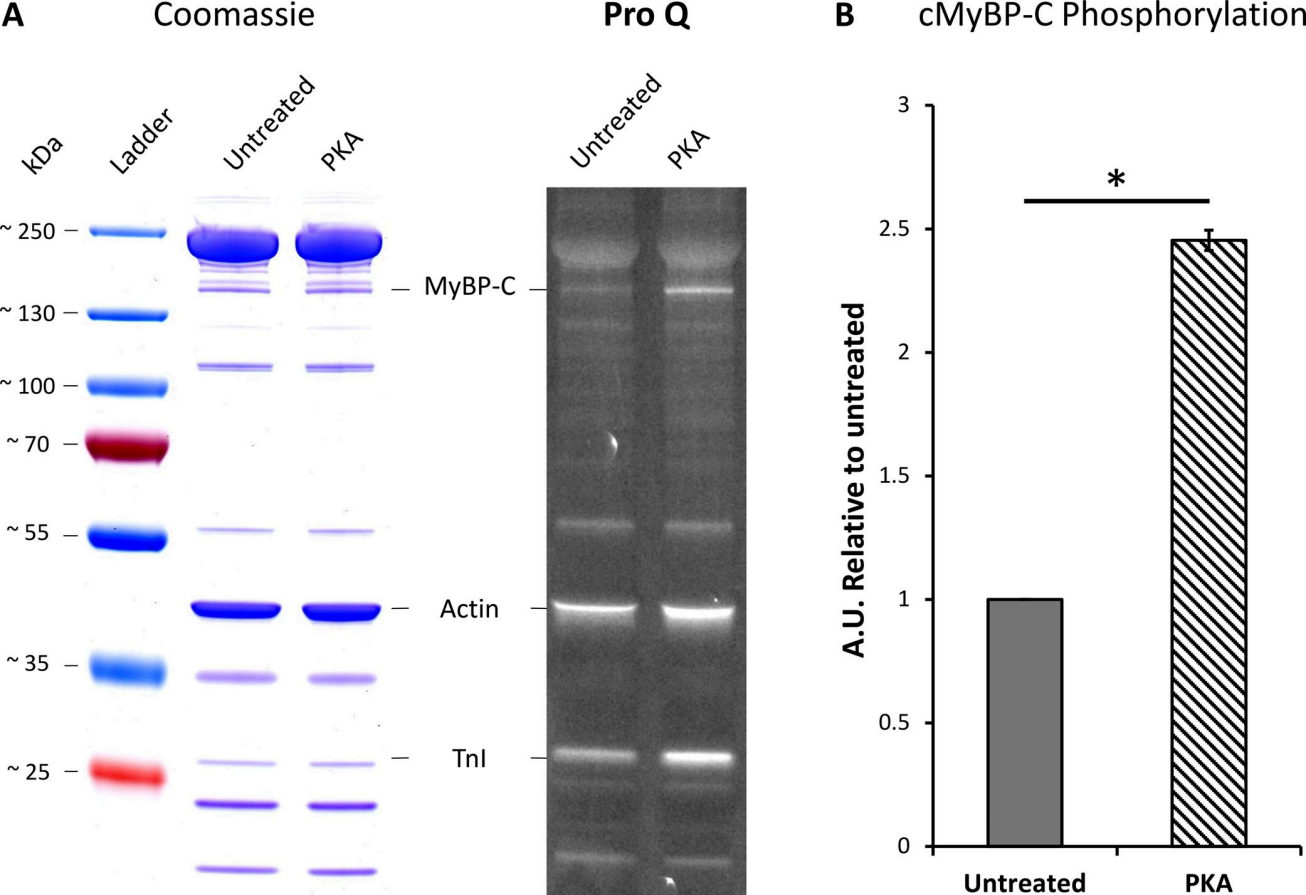
**Quantification of cMyBP-C phosphorylation after PKA treatment of porcine cardiac myofibrils.** Protein was isolated from PKA-treated myofibrils and compared with untreated myofibrils. **(A)** Samples were run on a 4–20% gradient SDS-PAGE gel and stained with Pro-Q Diamond Phosphoprotein Gel Stain (right) followed by Coomassie blue stain (left) to quantify total protein. **(B)** Densitometry analysis was performed on the cMyBP-C Pro-Q bands normalized to the same band in the Coomassie gel, and cMyBP-C treatment with PKA results in a ∼2.5-fold increase in phosphorylation relative to the untreated group (*N* = 1, *n* = 2). Data shown are the mean ± SEM. Significance was calculated with a one-tailed *t* test, *P = 0.025. Source data are available for this figure: SourceData F3.

With an understanding of the untreated porcine cardiac myofibril established, we then used PKA to reproduce the effects of β-adrenergic stimulation of cardiac muscle. The main targets for PKA are cMyBP-C, cTnI, and Titin (Yamasaki et al., 2002; Krüger and Linke, 2006; Ahmed and Lindsey, 2009). Using the Pro-Q phosphorylation gel stain, we found that treatment with PKA led to a statistically significant, 2.5-fold increase in phosphorylation of cMyBP-C (Fig. 3). cTnI was also phosphorylated after PKA treatment; however, because cTnI does not impact the system under relaxed conditions, we did not study it further. Examining the change in percent SRX by fitting the cumulative residence time histogram (Fig. 1 D) for Cy3-ATP lifetimes revealed PKA phosphorylation had no significant effect on myosin activity when measured across all zones (46.2% versus 46.3% [95% CI (52.7, 41.7)]). However, more detailed analysis of the distribution of myosin activity (see Fig. 2 and Table 1) within the sarcomere thick filament indicated, relative to untreated myofibrils, the absolute SRX population after PKA phosphorylation was significantly reduced by 16.3% in the C-zone (to 37.4% [95% CI {43.8, 31.3}]), consistent with PKA phosphorylating cMyBP-C and derepressing myosin activity. A significant decrease in the absolute SRX population was also identified in the P-zone (to 46.5% [95% CI {53.3, 39.3}]). Surprisingly, the absolute population of SRX significantly increased by 14.9% in the D-zone (to 59.0% (95% CI [73.9, 50.1]), compensating for the decreases in the P- and C-zones. These results explain the lack of a change in the SRX population across the whole sarcomere between untreated and PKA phosphorylated myofibrils.

### HCM-linked cMyBP-C haploinsufficiency enhances the DRX population of myosin heads specifically in the C-zone

**Figure 4.**
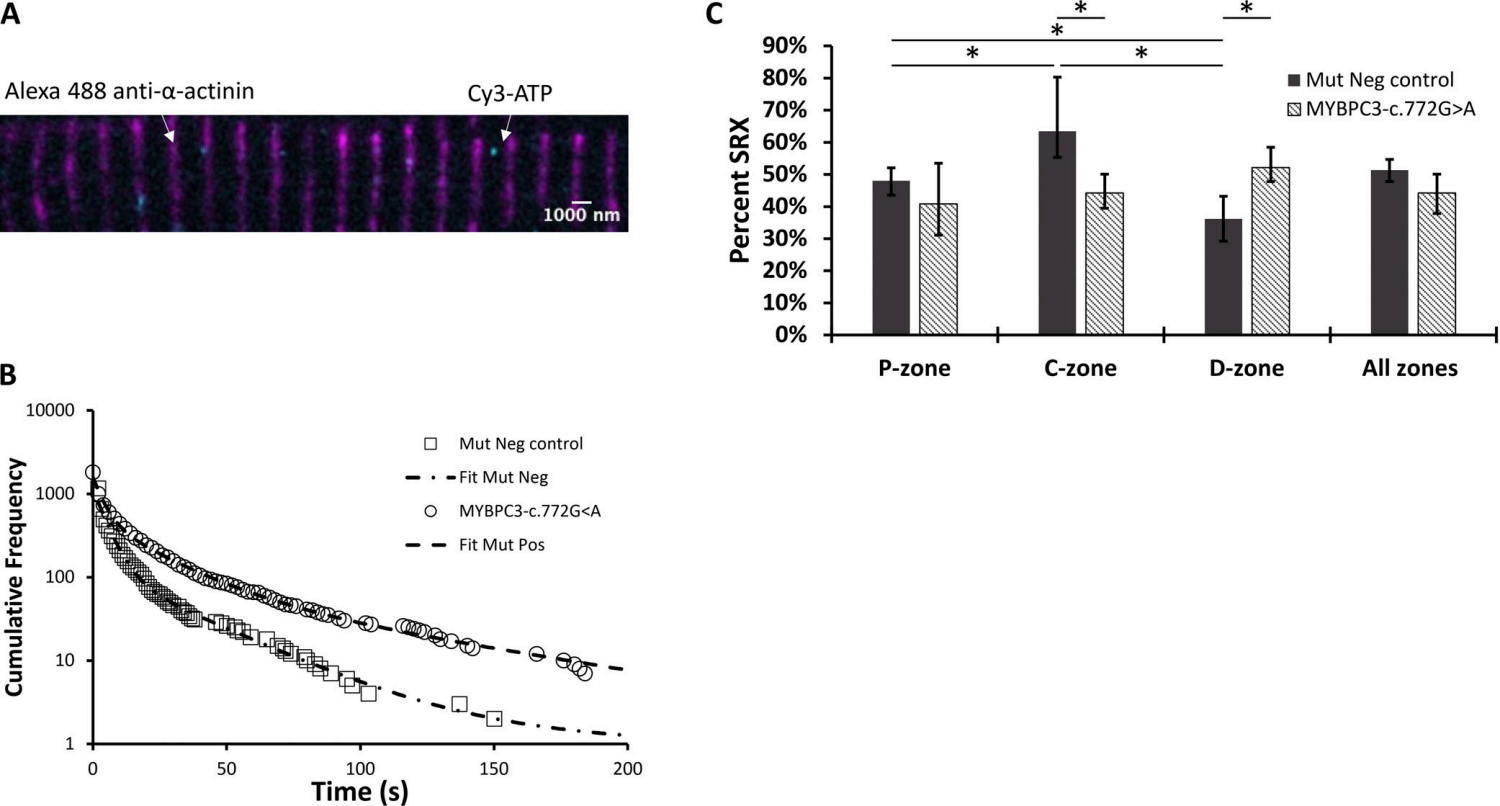
***MYBPC3*-c.772G>A mutation alters regulation of the thick filament by accelerating ATP turnover in the C-zone of the sarcomere. (A)** Representative image of a human cardiac myofibril illustrating 561-nm illuminated Cy3-ATP (cyan) and 488-nm illuminated α-actinin–containing Z-disks (magenta). **(B)** Normalized cumulative residence time histogram for all ATP binding events across the entire thick filament for mutation-negative control (squares; *N* = 2, *n* = 1,161 events) and *MYBPC3*-c.772G>A human ventricular myofibrils (circles; *N* = 1, *n* = 1,813 events). Dashed lines describe the cumulative exponential fits for both datasets. Cumulative frequency, y axis, is plotted on a logarithmic scale. **(C)** Percentage of myosin heads quantified to be in the SRX biochemical state in mutation-negative control human myectomy myofibrils and HCM *MYBPC3*-c.772G>A human myectomy myofibrils. The plot includes all zones and subsarcomeric zones of the thick filament. Data are shown as the mean and 95% CI errors, with significance calculated using bootstrap analysis and indicated by *. Table 2 provides the values used in the graph.

Using the same high-resolution single-molecule methods, we also analyzed the distribution of myosin activity within myofibrils isolated from human surgical myectomy samples that were either mutation-negative for HCM (“mutation neg control”) or mutation-positive for HCM (*MYBPC3*-c.772G>A) (Fig. 4 A). There was no significant difference in average sarcomere length between the two groups (mutation neg control = 1.74 ± 0.11 μm SD, *MYBPC3*-c.772G>A = 1.75 ± 0.10 μm SD). A cumulative residence time histogram was used to fit both datasets (Fig. 4 B), and the cumulative frequency decay was described again by three-exponential fits. For the mutation-negative control, the relative populations of SRX myosin heads revealed remarkable consistency with our above results from untreated porcine myofibrils. The percent population of SRX myosins in human control myofibrils was 51.3% (95% CI [54.7, 47.8]) across the entire thick filament, and not significantly different from porcine. Subdividing our tracked ATP binding lifetimes into the P-, C-, and D-zones revealed 48.0% (95% CI [52, 43.5]) SRX in the P-zone, 63.4% (95% CI [80.3, 55.3]) SRX in the C-zone, and 36.2% (95% CI [43.2, 29.1]) SRX in the D-zone (Fig. 4 C and summarized in Table 2).

**Table 2.** Values for the percentage change in the SRX population across the thick filament for the MYBPC3-c.772G<A mutation and a mutation negative control in human cardiac myofibrils, as plotted in Fig. 4

	% SRX			
	P	C	D	All zones
Mut neg control [95% CI]	**48** [52, 43.5]	**63.4** [80.3, 55.3]	**36.2** [43.2, 29.1]	**51.3** [54.7, 47.8]
*n*	*285*	*341*	*535*	*1,161*
MYBPC3-c.772G<A [95% CI]	**40.8** [53.5, 31.1]	**44.3** [50, 39.5]	**52.2** [58.5, 47.8]	**44.2** [50, 37.8]
*n*	*404*	*591*	*818*	*1,813*
Absolute Δ SRX (%) [95% CI]	**−7.2** [6.4, −18.2]	**−19.2** [-9, −38.4]	**16** [24.7, 7.2]	**−7.1** [-0.3, −14.7]

Measured values for the percentage SRX are given in bold and 95% CI in square brackets. The absolute difference in SRX as a result of PKA treatment relative to untreated myofibrils is given in the bottom row. The number of events (n) are shown in italics for each area of the thick filament and include nonspecific ATP binding events.

Above, we have shown that elevated cMyBP-C phosphorylation increases myosin activity in the thick filament. cMyBP-C haploinsufficiency has previously been shown to also increase myosin activity (McNamara et al., 2016; Toepfer et al., 2019); therefore, we used our spatially explicit single-molecule approach to investigate the effects of cMyBP-C haploinsufficiency caused by a HCM-linked mutation in *MYBPC3* (*MYBPC3*-c.772G>A) (Pioner et al., 2023). When averaged across the entire thick filament, there was no statistical significance as a result of the HCM mutation in the absolute percentage of SRX heads compared with mutation-negative control samples (44.2% versus 51.3% [95% CI {54.7, 47.8}]), respectively, Fig. 4 C). However, when our Cy3-ATP imaging results were analyzed by subsarcomeric zones of the thick filament, more nuanced differences emerged. Compared with the mutation-negative control myofibrils, myofibrils with the *MYBPC3*-c.772G>A HCM mutation exhibited a significant 19% absolute decrease in SRX myosins in the C-zone (63.4–44.3% [95% CI {50, 39.5}]) accompanied by a significant 16% absolute increase in SRX myosins in the D-zone (36.2–52.2% [95% CI {58.5, 47.8}]) (Fig. 4 C). These zonal changes were not accompanied by a statistically significant change to the SRX population in the P-zone. The opposing changes between the C- and D-zones in the presence of this *MYBPC3* HCM mutation help explain why no significant change was observed across the entire thick filament when compared to the mutation-negative control samples. However, the similarity in the compensatory change from the D-zone in the human mutation and porcine PKA-treated myofibrils provides two examples of negative cooperativity along the thick filament.

## Discussion

Cardiac muscle contraction is controlled on a beat-to-beat basis by the release and sequestration of calcium ions. However, the heart also requires a more nuanced response to external factors such as load and β-adrenergic stimulation. These affect both the timing of contraction and the force produced. Myosin motors generate force in the extremely dense environment of proteins arranged along the thick and thin filaments within cardiac myofibrils. By altering myosin’s ability to interact with actin, the level of force can be modulated. This is thick filament regulation, and is thought to occur through myosin occupying either a DRX state with a greater, or a SRX state with a lower, probability of interacting with actin. These states are characterized by their ATPase activities, with the DRX state being more catalytically active than the SRX state. The precise conformational nature and location of these myosin states along the thick filament are not yet known. The results from this study show that PKA phosphorylation of cMyBP-C and cMyBP-C haploinsufficiency release heads from the lower activity state preferentially in the cMyBP-C–containing C-zone of the sarcomere. Under both conditions, the D-zone alters its SRX population to compensate, suggesting potential negative cooperativity between the adjacent zones. Such an observation was not seen in skeletal muscle (Nelson et al., 2020; Pilagov et al., 2023), suggesting that cardiac ventricular muscle from both porcine and human sources may possess modified mechanisms of thick filament regulation. Importantly, these results also highlight the necessity of studying thick filament regulation using high-resolution methods provided by single-molecule, subsarcomeric imaging.

### cMyBP-C phosphorylation reduces myosin activity in the D-zone and derepresses in the C-zone

β-adrenergic stimulation leads to the activation of PKA, which has a few known targets in the sarcomere. One of these, cMyBP-C, has three to four primary serine phosphorylation sites in the N-terminal M-domain but also elsewhere (Jia et al., 2010; Ponnam et al., 2019; McNamara et al., 2019; Doh et al., 2022; Mamidi et al., 2017; Kooij et al., 2013), which are thought to affect the interactions of cMyBP-C with both myosin and actin (Mamidi et al., 2017; McNamara et al., 2019; Shaffer et al., 2009). In this study, we report that across the thick filament there is no overall change in the DRX population of myosins after PKA treatment. This result is unexpected since there is evidence showing an increase in myosin activity as a result of phosphorylation (Tong et al., 2008; Stelzer et al., 2007). The main difference is the effect of load, which is thought to differentially alter the activity of myosins along the thick filament (Brunello et al., 2020). This is in accord with this study where we found that when analyzed by zone, the thick filament’s C-zone exhibited a large increase in DRX myosins, consistent with phosphorylation of cMyBP-C derepressing the ATP turnover rates of myosin, due to reduced interaction between cMyBP-C and myosin (Colson et al., 2008; McNamara et al., 2019). This effect of PKA phosphorylation in the C-zone was accompanied by a decrease in myosin activity in the D-zone. Such a negatively cooperative response from the D-zone effectively rebalances the energy consumption across the whole thick filament. The origin of this cooperativity was unexpected and suggests additional unknown mechanisms of myosin regulation exist in the D-zone. However, in reverse, positive cooperativity has been seen in previous single-molecule studies of mouse cardiac muscle expressing α-myosin (Nelson et al., 2023). Similarly, in our recent single-molecule study of PKA phosphorylation using rabbit fast skeletal myofibrils (Pilagov et al., 2023), we also found a positively cooperative response between the two zones. Together, these results suggest that the β-cardiac system, present in both porcine and human cardiac myofibrils, behaves distinctly from other isoforms, a question that is of considerable importance to address in future studies. One possible origin of cooperativity is the physical interaction between myosin crowns along the thick filament backbone as suggested by high-resolution structural studies (Tamborrini et al., 2023; Dutta et al., 2024; Chen et al., 2024). However, alternative mechanisms for mediating cross-talk between the C- and D-zones must also be considered. One such factor could be titin phosphorylation (Yamasaki et al., 2002; Krüger and Linke, 2006; Hamdani et al., 2017), which may play a role in changing the ratio of DRX to SRX myosins within regions of the thick filament. PKA phosphorylation of titin reduces passive muscle tension (Yamasaki et al., 2002; Krüger and Linke, 2006) through the unfolding of the elastic N2B-cardiac unique sequence (N2Bus) region of titin, proximal to the Z-disk. Since we did not physically extend myofibrils during the single-molecule experiments, we observed sarcomere lengths of ∼1.76 µm for both untreated and PKA-treated porcine myofibrils (Fig. S2), resulting in near-complete overlap and loss of the I-band. Therefore, even though the N2Bus region of titin is located toward the Z-disk, this is where the D-zone would reside in our preparations, raising the possibility of an interaction between these proteins. In either case, investigations of the relative abundance of the DRX and SRX states in the zones of cardiac A-band require investigation in β-cardiac models engineered to possess mutations that directly address these potential mechanisms.

### A cMyBP-C*–*depleting HCM mutation also results in opposing zone-specific changes that offset the energy deficit

The critical role of cMyBP-C in maintaining native thick filament regulation is underscored by the fact that 40% of pathogenic/likely pathogenic HCM mutations occur in the *MYBPC3* gene (Marian, 2021). For many *MYBPC3* HCM mutations, their impact is manifested as a reduction in the amount of cMyBP-C (i.e., haploinsufficiency), which disrupts the regulation of cross-bridge cycling (Carrier et al., 2015; Barefield et al., 2015; Glazier et al., 2019; Suay-Corredera and Alegre-Cebollada, 2022; Pioner et al., 2023; Steczina et al., 2024). By analyzing human myofibrils from a patient with a haploinsufficiency-causing HCM mutation and comparing them with myofibrils from mutation-negative control patients, we have been able to directly assess how the HCM mutation alters myosin states across the thick filament.

When the native stoichiometry of sarcomeric proteins is disrupted by the *MYBPC3*-c.772G>A HCM mutation, significant acceleration of myosin ATPase activity was seen in the C-zone. In contrast, the D-zone saw a decrease in myosin activity mirroring the results from porcine cardiac myofibrils following PKA treatment. This may indicate that the energy usage imbalance created by the mutation leads to mitigating changes in the thick filament. Notably, human myectomy tissue with the *MYBPC3*-c.772G>A mutation exhibited significantly reduced cMyBP-C phosphorylation levels compared with donor tissue samples (Pioner et al., 2023). This suggests that despite compensation by the heart to offset increased myosin activity, cMyBP-C haploinsufficiency predominates and requires changes in the D-zone as well. As discussed above, this negatively cooperative response between the two adjacent zones appears to be specific to β-myosin heavy chain–expressing cardiac tissue and the root of this adaptive response will be explored in future studies.

### Conclusions

This study provides new insights into the zone-specific regulation of myosin activity in β-myosin heavy chain–expressing cardiac tissue, highlighting the substantial influence cMyBP-C has on myosin activity. Using high-resolution, single-molecule imaging, we demonstrated that both PKA-induced phosphorylation of cMyBP-C and cMyBP-C haploinsufficiency exercise their effects by increasing myosin activity primarily in the C-zone. Our results have also shown a compensatory decrease in myosin activity in the D-zone of the sarcomere under both conditions of altered cMyBP-C, which suggests a unique negative cooperativity mechanism not observed in skeletal or mouse cardiac muscle. These findings emphasize the distinct regulatory properties of β-cardiac myosin compared with other isoforms, with potential implications for understanding the pathophysiology of *MYBPC3*-linked HCM and the development of targeted therapies.

## Supplementary Material

Table S1 provides the fitted parameters and errors.

SourceData F3 is the source file for Fig. 3.

## Data Availability

Data are available from the authors upon reasonable request. Coding of custom scripts is available here: https://github.com/Kad-Lab/Myofibril-phosphorylation.

## References

[bib1] Ahmed, S.H., and M.L. Lindsey. 2009. Titin phosphorylation: Myocardial passive stiffness regulated by the intracellular giant. Circ. Res. 105:611–613. 10.1161/CIRCRESAHA.109.206912 19797191 PMC2765037

[bib2] Amrute-Nayak, M., K.-A. Lambeck, A. Radocaj, H.E. Huhnt, T. Scholz, N. Hahn, G. Tsiavaliaris, W.J. Walter, and B. Brenner. 2014. ATP turnover by individual myosin molecules hints at two conformers of the myosin active site. Proc. Natl. Acad. Sci. USA. 111:2536–2541. 10.1073/pnas.1316390111 24550279 PMC3932867

[bib3] Barefield, D., M. Kumar, J. Gorham, J.G. Seidman, C.E. Seidman, P.P. de Tombe, and S. Sadayappan. 2015. Haploinsufficiency of MYBPC3 exacerbates the development of hypertrophic cardiomyopathy in heterozygous mice. J. Mol. Cell. Cardiol. 79:234–243. 10.1016/j.yjmcc.2014.11.018 25463273 PMC4642280

[bib4] Bennett, P.M., and M. Gautel. 1996. Titin domain patterns correlate with the axial disposition of myosin at the end of the thick filament. J. Mol. Biol. 259:896–903. 10.1006/jmbi.1996.0367 8683592

[bib5] Bremel, R.D., and A. Weber. 1975. Calcium binding to rabbit skeletal myosin under physiological conditions. Biochim. Biophys. Acta. 376:366–374. 10.1016/0005-2728(75)90028-6 1115783

[bib6] Brunello, E., L. Fusi, A. Ghisleni, S.-J. Park-Holohan, J.G. Ovejero, T. Narayanan, and M. Irving. 2020. Myosin filament-based regulation of the dynamics of contraction in heart muscle. Proc. Natl. Acad. Sci. USA. 117:8177–8186. 10.1073/pnas.1920632117 32220962 PMC7149498

[bib7] Carrier, L., G. Mearini, K. Stathopoulou, and F. Cuello. 2015. Cardiac myosin-binding protein C (MYBPC3) in cardiac pathophysiology. Gene. 573:188–197. 10.1016/j.gene.2015.09.008 26358504 PMC6660134

[bib8] Chen, L., J. Liu, H. Rastegarpouyani, P.M.L. Janssen, J.R. Pinto, and K.A. Taylor. 2024. Structure of mavacamten-free human cardiac thick filaments within the sarcomere by cryoelectron tomography. Proc. Natl. Acad. Sci. USA. 121:e2311883121. 10.1073/pnas.2311883121 38386705 PMC10907299

[bib9] Colson, B.A., T. Bekyarova, D.P. Fitzsimons, T.C. Irving, and R.L. Moss. 2007. Radial displacement of myosin cross-bridges in mouse myocardium due to ablation of myosin binding protein-C. J. Mol. Biol. 367:36–41. 10.1016/j.jmb.2006.12.063 17254601 PMC1892277

[bib10] Colson, B.A., T. Bekyarova, M.R. Locher, D.P. Fitzsimons, T.C. Irving, and R.L. Moss. 2008. Protein kinase A-mediated phosphorylation of Cmybp-c increases proximity of myosin heads to actin in resting myocardium. Circ. Res. 103:244–251. 10.1161/CIRCRESAHA.108.178996 18599866 PMC2810832

[bib11] Colson, B.A., M.R. Locher, T. Bekyarova, J.R. Patel, D.P. Fitzsimons, T.C. Irving, and R.L. Moss. 2010. Differential roles of regulatory light chain and myosin binding protein-C phosphorylations in the modulation of cardiac force development. J. Physiol. 588:981–993. 10.1113/jphysiol.2009.183897 20123786 PMC2849963

[bib12] Craig, R., and G. Offer. 1976. The location of C protein in rabbit skeletal muscle. Proc. R. Soc. Lond. B Biol. Sci. 192:451–461. 10.1098/rspb.1976.0023 4802

[bib13] Desai, R., M.A. Geeves, and N.M. Kad. 2015. Using fluorescent myosin to directly visualize cooperative activation of thin filaments. J. Biol. Chem. 290:1915–1925. 10.1074/jbc.M114.609743 25429108 PMC4303648

[bib14] Descriptor_based_registration . 2015. Github. https://github.com/fiji/Descriptor_based_registration (accessed July 11, 2023).

[bib15] Doh, C.Y., K.L. Dominic, C.E. Swanberg, N. Bharambe, B.B. Willard, L. Li, R. Ramachandran, and J.E. Stelzer. 2022. Identification of phosphorylation and other post-translational modifications in the central C4C5 domains of murine cardiac myosin binding protein C. ACS Omega. 7:14189–14202. 10.1021/acsomega.2c00799 35573219 PMC9089392

[bib16] Dutta, D., Y. Kim, J.G. Seidman, R. Craig, C.E. Seidman, and R. Padron. 2024. Pathogenic cardiac thick filament variants: A structural perspective. Biophys. J. 123:403a. 10.1016/j.bpj.2023.11.2468

[bib17] Ershov, D., M.-S. Phan, J.W. Pylvänäinen, S.U. Rigaud, L. Le Blanc, A. Charles-Orszag, J.R.W. Conway, R.F. Laine, N.H. Roy, D. Bonazzi, . 2022. TrackMate 7: Integrating state-of-the-art segmentation algorithms into tracking pipelines. Nat. Methods. 19:829–832. 10.1038/s41592-022-01507-1 35654950

[bib18] Fusi, L., E. Brunello, Z. Yan, and M. Irving. 2016. Thick filament mechano-sensing is a calcium-independent regulatory mechanism in skeletal muscle. Nat. Commun. 7:13281. 10.1038/ncomms13281 27796302 PMC5095582

[bib19] Gelles, J., B.J. Schnapp, and M.P. Sheetz. 1988. Tracking kinesin-driven movements with nanometre-scale precision. Nature. 331:450–453. 10.1038/331450a0 3123999

[bib20] Glazier, A.A., A. Thompson, and S.M. Day. 2019. Allelic imbalance and haploinsufficiency in MYBPC3-linked hypertrophic cardiomyopathy. Pflugers Arch. 471:781–793. 10.1007/s00424-018-2226-9 30456444 PMC6476680

[bib21] Gordon, A.M., E. Homsher, and M. Regnier. 2000. Regulation of contraction in striated muscle. Physiol. Rev. 80:853–924. 10.1152/physrev.2000.80.2.853 10747208

[bib22] Hamdani, N., M. Herwig, and W.A. Linke. 2017. Tampering with springs: Phosphorylation of titin affecting the mechanical function of cardiomyocytes. Biophys. Rev. 9:225–237. 10.1007/s12551-017-0263-9 28510118 PMC5498327

[bib23] Heling, L.W.H.J., M.A. Geeves, and N.M. Kad. 2020. MyBP-C: One protein to govern them all. J. Muscle Res. Cell Motil. 41:91–101. 10.1007/s10974-019-09567-1 31960266 PMC7109175

[bib24] Hooijman, P., M.A. Stewart, and R. Cooke. 2011. A new state of cardiac myosin with very slow ATP turnover: A potential cardioprotective mechanism in the heart. Biophys. J. 100:1969–1976. 10.1016/j.bpj.2011.02.061 21504733 PMC3077696

[bib25] Jia, W., J.F. Shaffer, S.P. Harris, and J.A. Leary. 2010. Identification of novel protein kinase A phosphorylation sites in the M-domain of human and murine cardiac myosin binding protein-C using mass spectrometry analysis. J. Proteome Res. 9:1843–1853. 10.1021/pr901006h 20151718 PMC3000796

[bib26] Konno, T., S. Chang, J.G. Seidman, and C.E. Seidman. 2010. Genetics of hypertrophic cardiomyopathy. Curr. Opin. Cardiol. 25:205–209. 10.1097/HCO.0b013e3283375698 20124998 PMC2932754

[bib27] Kooij, V., R.J. Holewinski, A.M. Murphy, and J.E. Van Eyk. 2013. Characterization of the cardiac myosin binding protein-C phosphoproteome in healthy and failing human hearts. J. Mol. Cell. Cardiol. 60:116–120. 10.1016/j.yjmcc.2013.04.012 23619294 PMC3710717

[bib28] Krüger, M., and W.A. Linke. 2006. Protein kinase-A phosphorylates titin in human heart muscle and reduces myofibrillar passive tension. J. Muscle Res. Cell Motil. 27:435–444. 10.1007/s10974-006-9090-5 16897574

[bib29] Lehman, W., J. Kendrick-Jones, and A.G. Szent-Györgyi. 1973. Myosin-linked regulatory systems: Comparative studies. Cold Spring Harb. Symp. Quant. 37:319–330. 10.1101/SQB.1973.037.01.042

[bib30] Levine, R.J., R.W. Kensler, Z. Yang, J.T. Stull, and H.L. Sweeney. 1996. Myosin light chain phosphorylation affects the structure of rabbit skeletal muscle thick filaments. Biophys. J. 71:898–907. 10.1016/S0006-3495(96)79293-7 8842229 PMC1233547

[bib31] Linari, M., E. Brunello, M. Reconditi, L. Fusi, M. Caremani, T. Narayanan, G. Piazzesi, V. Lombardi, and M. Irving. 2015. Force generation by skeletal muscle is controlled by mechanosensing in myosin filaments. Nature. 528:276–279. 10.1038/nature15727 26560032

[bib32] Ma, W., S. Nag, H. Gong, L. Qi, and T.C. Irving. 2022. Cardiac myosin filaments are directly regulated by calcium. J. Gen. Physiol. 154:e202213213. 10.1085/jgp.202213213 36327149 PMC9629851

[bib33] Mamidi, R., K.S. Gresham, J. Li, and J.E. Stelzer. 2017. Cardiac myosin binding protein-C Ser^302^ phosphorylation regulates cardiac β-adrenergic reserve. Sci. Adv. 3:e1602445. 10.1126/sciadv.1602445 28345052 PMC5345928

[bib34] Marian, A.J. 2021. Molecular genetic basis of hypertrophic cardiomyopathy. Circ. Res. 128:1533–1553. 10.1161/CIRCRESAHA.121.318346 33983830 PMC8127615

[bib35] McNamara, J.W., A. Li, N.J. Smith, S. Lal, R.M. Graham, K.B. Kooiker, S.J. van Dijk, C.G.D. Remedios, S.P. Harris, and R. Cooke. 2016. Ablation of cardiac myosin binding protein-C disrupts the super-relaxed state of myosin in murine cardiomyocytes. J. Mol. Cell. Cardiol. 94:65–71. 10.1016/j.yjmcc.2016.03.009 27021517 PMC4861668

[bib36] McNamara, J.W., R.R. Singh, and S. Sadayappan. 2019. Cardiac myosin binding protein-C phosphorylation regulates the super-relaxed state of myosin. Proc. Natl. Acad. Sci. USA. 116:11731–11736. 10.1073/pnas.1821660116 31142654 PMC6575167

[bib37] Mohran, S., K. Kooiker, M. Mahoney-Schaefer, C. Mandrycky, K. Kao, A.-Y. Tu, J. Freeman, F. Moussavi-Harami, M. Geeves, and M. Regnier. 2024. The biochemically defined super relaxed state of myosin-A paradox. J. Biol. Chem. 300:105565. 10.1016/j.jbc.2023.105565 38103642 PMC10819765

[bib38] Nelson, S., S. Beck-Previs, S. Sadayappan, C. Tong, and D.M. Warshaw. 2023. Myosin-binding protein C stabilizes, but is not the sole determinant of SRX myosin in cardiac muscle. J. Gen. Physiol. 155:e202213276. 10.1085/jgp.202213276 36688870 PMC9884578

[bib39] Nelson, S. R., A. Li, S. Beck-Previs, G.G. Kennedy, and D.M. Warshaw. 2020. Imaging ATP consumption in resting skeletal muscle: One molecule at a time. Biophys. J. 119:1050–1055. 10.1016/j.bpj.2020.07.036 32857963 PMC7499091

[bib40] Nogara, L., N. Naber, E. Pate, M. Canton, C. Reggiani, and R. Cooke. 2016. Piperine’s mitigation of obesity and diabetes can be explained by its up-regulation of the metabolic rate of resting muscle. Proc. Natl. Acad. Sci. USA. 113:13009–13014. 10.1073/pnas.1607536113 27799519 PMC5135373

[bib41] Pilagov, M., L.W.H.J. Heling, J. Walklate, M.A. Geeves, and N.M. Kad. 2023. Single-molecule imaging reveals how mavacamten and PKA modulate ATP turnover in skeletal muscle myofibrils. J. Gen. Physiol. 155:e202213087. 10.1085/jgp.202213087 36394553 PMC9674027

[bib42] Pioner, J.M., G. Vitale, S. Steczina, M. Langione, F. Margara, L. Santini, F. Giardini, E. Lazzeri, N. Piroddi, B. Scellini, . 2023. Slower calcium handling balances faster cross-bridge cycling in human MYBPC3 HCM. Circ. Res. 132:628–644. 10.1161/circresaha.122.321956 36744470 PMC9977265

[bib43] Podlubnaya, Z.A., I. Kakol, A. Moczarska, D. Stepkowski, and S. Udaltsov. 2000a. Truncation of vertebrate striated muscle myosin light chains disturbs calcium-induced structural transitions in synthetic myosin filaments. J. Struct. Biol. 131:225–233. 10.1006/jsbi.2000.4265 11052895

[bib44] Podlubnaya, Z.A., S.L. Malyshev, K. Nieznański, and D. Stepkowski. 2000b. Order-disorder structural transitions in synthetic filaments of fast and slow skeletal muscle myosins under relaxing and activating conditions. Acta Biochim. Pol. 47:1007–1017. 11996091

[bib45] Podlubnaya, Z., I. Kakol, A. Moczarska, D. Stepkowski, and S. Udaltsov. 1999. Calcium-induced structural changes in synthetic myosin filaments of vertebrate striated muscles. J. Struct. Biol. 127:1–15. 10.1006/jsbi.1999.4129 10479612

[bib46] Ponnam, S., I. Sevrieva, Y.-B. Sun, M. Irving, and T. Kampourakis. 2019. Site-specific phosphorylation of myosin binding protein-C coordinates thin and thick filament activation in cardiac muscle. Proc. Natl. Acad. Sci. USA. 116:15485–15494. 10.1073/pnas.1903033116 31308242 PMC6681757

[bib47] Previs, M.J., J.Y. Mun, A.J. Michalek, S.B. Previs, J. Gulick, J. Robbins, D.M. Warshaw, and R. Craig. 2016. Phosphorylation and calcium antagonistically tune myosin-binding protein C’s structure and function. Proc. Natl. Acad. Sci. USA. 113:3239–3244. 10.1073/pnas.1522236113 26908872 PMC4812749

[bib48] Shaffer, J.F., R.W. Kensler, and S.P. Harris. 2009. The myosin-binding protein C motif binds to F-actin in a phosphorylation-sensitive manner. J. Biol. Chem. 284:12318–12327. 10.1074/jbc.M808850200 19269976 PMC2673300

[bib49] Steczina, S., S. Mohran, L.R.J. Bailey, T.S. McMillen, K.B. Kooiker, N.B. Wood, J. Davis, M.J. Previs, I. Olivotto, J.M. Pioner, . 2024. MYBPC3-c.772G>A mutation results in haploinsufficiency and altered myosin cycling kinetics in a patient induced stem cell derived cardiomyocyte model of hypertrophic cardiomyopathy. J. Mol. Cell. Cardiol. 191:27–39. 10.1016/j.yjmcc.2024.04.010 38648963 PMC11116032

[bib50] Stelzer, J.E., J.R. Patel, J.W. Walker, and R.L. Moss. 2007. Differential roles of cardiac myosin-binding protein C and cardiac troponin I in the myofibrillar force responses to protein kinase A phosphorylation. Circ. Res. 101:503–511. 10.1161/CIRCRESAHA.107.153650 17641226

[bib51] Stewart, M.A., K. Franks-Skiba, S. Chen, and R. Cooke. 2010. Myosin ATP turnover rate is a mechanism involved in thermogenesis in resting skeletal muscle fibers. Proc. Natl. Acad. Sci. USA. 107:430–435. 10.1073/pnas.0909468107 19966283 PMC2806748

[bib52] Suay-Corredera, C., and J. Alegre-Cebollada. 2022. The mechanics of the heart: Zooming in on hypertrophic cardiomyopathy and CMyBP-C. FEBS Lett. 596:703–746. 10.1002/1873-3468.14301 35224729

[bib53] Tamborrini, D., Z. Wang, T. Wagner, S. Tacke, M. Stabrin, M. Grange, A.L. Kho, M. Rees, P. Bennett, M. Gautel, . 2023. Structure of the native myosin filament in the relaxed cardiac sarcomere. Nature. 623:863–871. 10.1038/s41586-023-06690-5 37914933 PMC10665186

[bib54] Tinevez, J.-Y., N. Perry, J. Schindelin, G.M. Hoopes, G.D. Reynolds, E. Laplantine, S.Y. Bednarek, S.L. Shorte, and K.W. Eliceiri. 2017. TrackMate: An open and extensible platform for single-particle tracking. Methods. 115:80–90. 10.1016/j.ymeth.2016.09.016 27713081

[bib55] Toepfer, C.N., H. Wakimoto, A.C. Garfinkel, B. McDonough, D. Liao, J. Jiang, A.C. Tai, J.M. Gorham, I.G. Lunde, M. Lun, . 2019. Hypertrophic cardiomyopathy mutations in MYBPC3 dysregulate myosin. Sci. Transl. Med. 11:eaat1199. 10.1126/scitranslmed.aat1199 30674652 PMC7184965

[bib56] Tong, C.W., J.E. Stelzer, M.L. Greaser, P.A. Powers, and R.L. Moss. 2008. Acceleration of crossbridge kinetics by protein kinase A phosphorylation of cardiac myosin binding protein C modulates cardiac function. Circ. Res. 103:974–982. 10.1161/CIRCRESAHA.108.177683 18802026 PMC2867079

[bib57] Tonino, P., B. Kiss, J. Gohlke, J.E. Smith 3rd, and H. Granzier. 2019. Fine mapping Titin’s C-Zone: Matching cardiac myosin-binding protein C Stripes with titin’s super-repeats. J. Mol. Cell. Cardiol. 133:47–56. 10.1016/j.yjmcc.2019.05.026 31158359 PMC6639027

[bib58] Toseland, C.P., and M.R. Webb. 2011. Fluorescent nucleoside triphosphates for single-molecule enzymology. Methods Mol. Biol. 778:161–174. 10.1007/978-1-61779-261-8_11 21809206

[bib59] Ušaj, M., L. Moretto, V. Vemula, A. Salhotra, and A. Månsson. 2021. Single molecule turnover of fluorescent ATP by myosin and actomyosin unveil elusive enzymatic mechanisms. Commun. Biol. 4:64. 10.1038/s42003-020-01574-0 33441912 PMC7806905

[bib60] Vikhorev, P.G., M.A. Ferenczi, and S.B. Marston. 2016. Instrumentation to study myofibril mechanics from static to artificial simulations of cardiac cycle. MethodsX. 3:156–170. 10.1016/j.mex.2016.02.006 27047763 PMC4796715

[bib61] Yamasaki, R., Y. Wu, M. McNabb, M. Greaser, S. Labeit, and H. Granzier. 2002. Protein kinase A phosphorylates titin’s cardiac-specific N2B domain and reduces passive tension in rat cardiac myocytes. Circ. Res. 90:1181–1188. 10.1161/01.res.0000021115.24712.99 12065321

