# Supplementary material for: Spatially resolving how cMyBP-C phosphorylation and haploinsufficiency in porcine and human myofibrils affect β-cardiac myosin activity
Source: J Gen Physiol. 2025 Jul 7;157(5):e202413628. doi: 10.1085/jgp.202413628 (PMC12232901; doi:10.1085/jgp.202413628)
Supplement: Table S1 — provides the fitted parameters and errors. [file jgp_202413628_tables1.docx]

**Table S1**

| Condition | Non-specific lifetime (s) | DRX lifetime (s) | SRX lifetime (s) |
| --- | --- | --- | --- |
| Untreated porcine | 1.36 [1.1, 1.5] | 10.29 [8.6, 10.8] | 59.9 [52.2, 61.3] |
| PKA-treated porcine | 1.68 [1.4,2.0] | 13.00 [11.1, 15.5] | 71.22 [56.4, 76.7] |
| SMN | ND* | 9.62 [8.0, 11.5] | 59.32 [54.8, 65.4] |
| SMP | 3.86 [3.2, 4.6] | 20.19 [17.4, 25.0] | 83.0 [72.7, 95.8] |

The data in this table are fitted rate constants for all zones uncorrected for photobleaching in all conditions studied. Numbers in [] are 95% confidence intervals.

*For this condition no appreciable faster component was detected.
