# Supplementary figures and images for: Spatially resolving how cMyBP-C phosphorylation and haploinsufficiency in porcine and human myofibrils affect β-cardiac myosin activity
Source: J Gen Physiol. 2025 Jul 7;157(5):e202413628. doi: 10.1085/jgp.202413628 (PMC12232901; doi:10.1085/jgp.202413628)

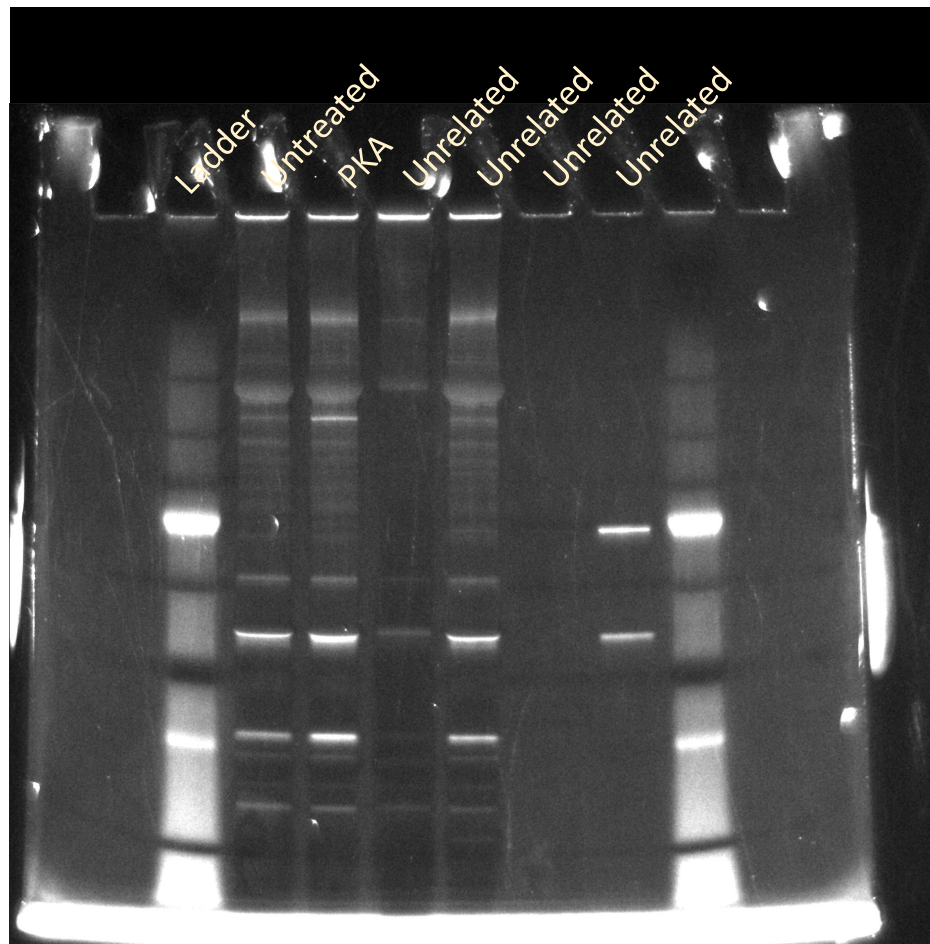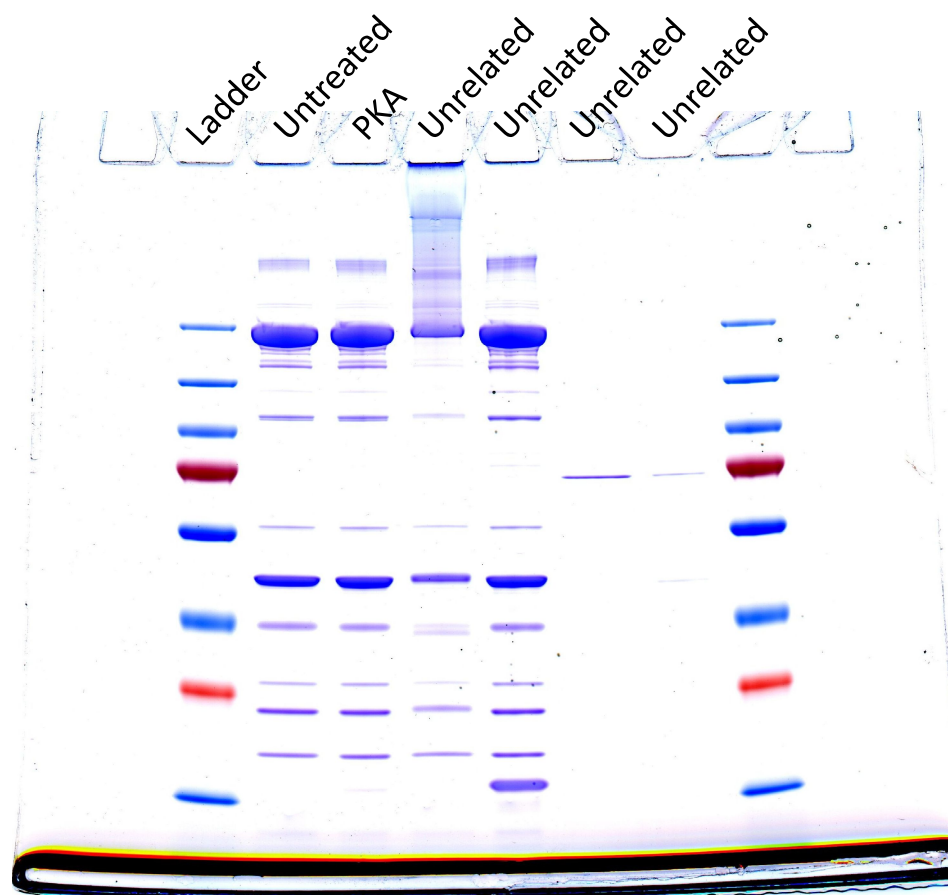

Supplement: SourceData F3 — is the source file for Fig. 3. [file jgp_202413628_sourcedataf3.pdf]
